# Where’s the evidence? a systematic review of economic analyses of residential aged care infrastructure

**DOI:** 10.1186/s12913-017-2165-8

**Published:** 2017-03-21

**Authors:** Tiffany Easton, Rachel Milte, Maria Crotty, Julie Ratcliffe

**Affiliations:** 1 Flinders Health Economics Group, School of Medicine, Flinders University, Adelaide, SA Australia; 2 NHMRC Partnership Centre on Dealing with Cognitive and Related Functional Decline in Older People, Canberra, Australia; 3 Rehabilitation, Aged and Extended Care, School of Health Sciences, Flinders University, GPO Box 2100, Adelaide, SA 5001 Australia; 4 Institute for Choice, Business School, University of South Australia, Adelaide, SA Australia

**Keywords:** Systematic review, Ageing, Long-term care, Infrastructure, Economic evaluation

## Abstract

**Background:**

Residential care infrastructure, in terms of the characteristics of the organisation (such as proprietary status, size, and location) and the physical environment, have been found to directly influence resident outcomes. This review aimed to summarise the existing literature of economic evaluations of residential care infrastructure.

**Methods:**

A systematic review of English language articles using AgeLine, CINAHL, Econlit, Informit (databases in Health; Business and Law; Social Sciences), Medline, ProQuest, Scopus, and Web of Science with retrieval up to 14 December 2015. The search strategy combined terms relating to nursing homes, economics, and older people. Full economic evaluations, partial economic evaluations, and randomised trials reporting more limited economic information, such as estimates of resource use or costs of interventions were included. Data was extracted using predefined data fields and synthesized in a narrative summary to address the stated review objective.

**Results:**

Fourteen studies containing an economic component were identified. None of the identified studies attempted to systematically link costs and outcomes in the form of a cost-benefit, cost-effectiveness, or cost-utility analysis. There was a wide variation in approaches taken for valuing the outcomes associated with differential residential care infrastructures: 8 studies utilized various clinical outcomes as proxies for the quality of care provided, and 2 focused on resident outcomes including agitation, quality of life, and the quality of care interactions. Only 2 studies included residents living with dementia.

**Conclusions:**

Robust economic evidence is needed to inform aged care facility design. Future research should focus on identifying appropriate and meaningful outcome measures that can be used at a service planning level, as well as the broader health benefits and cost-saving potential of different organisational and environmental characteristics in residential care.

**Trial registration:**

International Prospective Register of Systematic Reviews (PROSPERO) registration number CRD42015015977.

**Electronic supplementary material:**

The online version of this article (doi:10.1186/s12913-017-2165-8) contains supplementary material, which is available to authorized users.

## Background

In most Organisation for Economic Co-operation and Development (OECD) countries, aged care accounts for approximately 1 to 1.5% of gross domestic product (GDP) in terms of government funding alone [1], and on average roughly two-thirds of this funding is allocated to residential care (incorporating care homes, intermediate care facilities, skilled nursing facilities, nursing homes, residential aged care facilities, and residential homes) [2]. Despite the ongoing research and development of alternatives to residential care, including initiatives to enable older people to remain at home for as long as possible [3–6], the number of older people receiving care in a residential facility has continued to grow [2]. Residential care settings tend to cater for individuals living with higher levels of disability and care needs than those in alternative settings such as community care [7, 8]. For instance, it is estimated that over 50% of residents in residential care have a recorded diagnosis of dementia [7, 9]. Recent literature suggests that for people with dementia with high levels of physical dependence, residential care can be less costly to provide at a societal level than home-based care [10–12]. This is primarily because of the high informal care costs for society arising from time spent by family and friends on supervision and care in home based settings [10, 11].

The organisational environment or infrastructure is widely discussed in residential aged care settings, in terms of both characteristics of the organisation (such as proprietary status, size, and location), and the physical environment. Structural design choices in residential aged care have been found to directly influence resident outcomes [13]. Improvements in areas such as behaviour, function, well-being, and care outcomes have been linked to specialised environmental design interventions [14]. In contrast, higher rates of depressive symptoms have been linked to larger facilities as well as facilities located in non-urban areas [15].

Economic evaluation research is increasingly being used in the health and aged care sectors in an effort to promote efficiency in the design and delivery of services [16–19]. Health economic evaluation is defined as the comparative analysis of alternative interventions in terms of both their costs (resource use) and outcomes [20]. In an economic evaluation, costs are expressed in terms of the benefit received, typically in the form of an incremental cost-effectiveness ratio (ICER). For example, in a cost-utility analysis, results are presented as the cost per quality-adjusted life year (QALY) gained in which the unit of effect is a ‘year in full health’. Outcomes can also be measured in ‘natural’ units, such as life-years gained or improvements in cognitive functioning, which can be incorporated into a cost-effectiveness analysis. Governmental agencies in healthcare, such as the National Institute for Health and Care Excellence (NICE) and similar bodies around the world, require cost-effectiveness evidence in the form of incremental cost per QALY [21].

While economic evaluation is well established for the evaluation of health technologies and interventions, techniques for assessing the economic value of health or quality of life benefits from infrastructure are much less common and tend to vary widely in the methodologies applied [22, 23]. In addition, economic evaluations conducted with older populations with high rates of dementia or cognitive impairment tend to encounter methodological issues arising from the reduced ability of this population to provide informed consent [24] and self-report their own quality of life [25, 26]. The measurement and valuation of resident outcomes in a residential aged care setting is a complex undertaking due to the majority of residents living with cognitive impairment and dementia [7, 9, 27, 28] however fully appraising these effects is important for evidence-based policy making.

Recent projections estimate that long-term care spending in OECD countries will more than double on average over the next 50 years [1, 29]. Given the ageing of the population [1] and the substantial amount of current and future funding governments provide and are projected to provide for residential care [1, 29], research in this area is warranted. The main objective of this review was to provide a systematic and narrative summary of the existing literature of economic evaluations of residential aged care infrastructure.

## Methods

### Protocol and registration

This review was conducted in accordance with the Joanna Briggs Institute (JBI) guidance for the systematic review of economic evaluation evidence [30]. A protocol for this systematic review was registered with the PROSPERO International Prospective Register of Systematic Reviews on 30 January 2015 (http://www.crd.york.ac.uk/PROSPERO; registration number CRD42015015977).

### Eligibility criteria

Eligible studies included full economic evaluations (e.g. cost-effectiveness analyses, cost-utility analyses, cost-benefit analyses), partial economic evaluations (e.g. cost analyses, cost minimisation analyses, cost consequences analyses), and randomised trials reporting more limited information, such as estimates of resource use or costs of interventions, pertaining to organisational and environmental characteristics aimed at improving the quality of care for older adults in a residential aged care setting. *Organisational characteristics* related to the overall business structure of the aged care provider, and included attributes such as demographics, proprietary status, size, and affiliation. *Environmental characteristics* referred to the physical setting and included tangible attributes such as private rooms, access to outdoors, familiar home-like components, and secure units.

### Search and study selection

Eight electronic bibliographic databases were searched from inception to 8 October 2014, including AgeLine, the Cumulative Index of Nursing and Allied Health Literature (CINAHL), Econlit, Informit (databases in Health; Business and Law; Social Sciences), Medline, ProQuest, Scopus, and Web of Science. An update search was run on 14 December 2015.

The search strategies were developed and reviewed with the assistance of two Health Sciences Librarians with expertise in systematic reviews. The strategy combined terms relating to nursing homes, economics, and older people, limited to English language. No study design or date limits were imposed on the search. The full search strategy is available on PROSPERO (http://www.crd.york.ac.uk/PROSPEROFILES/15977_STRATEGY_20150030.pdf).

Due to the large number of results retrieved when searching the multidisciplinary database ProQuest, limits to source type (scholarly journals, reports, dissertations and theses, conference papers and proceedings, and working papers) were applied to this database that were not part of the original search strategy. Newspapers, trade journals, wire feeds, magazines, other sources, books, and encyclopaedias and reference works were excluded.

Titles and abstracts of studies retrieved were reviewed in full by the primary author (T.E.). A second reviewer (see Acknowledgements) independently screened 10% of the titles and abstracts (L.P.L). Full text reports were retrieved for all citations that appeared to meet the inclusion criteria. All full text reports retrieved were reviewed independently by the primary author and second reviewer (T.E. and L.P.L.). Disagreement was resolved through discussion and consultation with a third reviewer (R.M.). Reasons for excluding studies were documented. The reference lists of included studies were hand searched for additional studies by the primary author (T.E.).

### Data extraction

The JBI Data Extraction Form for Economic Evaluations was used to extract data from the included studies (http://joannabriggs.org/assets/docs/jbc/operations/dataExtractionForms/JBC_Form_DataE_EconEval.pdf) [31]. Standardised data items extracted included descriptive data about the study and analysis including (i) study population/participants, intervention, comparator(s) and outcomes; (ii) study methods including prices and currency used for costing, time period, sensitivity analyses and measures of resource use; (iii) study context (geographical, health care and broader service delivery setting and culture); (iv) analysis methods. Results for the resource use and/or cost and/or cost-effectiveness measures and the author conclusions were also extracted. The primary author (T.E.) extracted all data. Neither the study selection nor the data extraction was blinded.

### Risk of bias assessment

Critical appraisal of studies was undertaken using the JBI Critical Appraisal Checklist for Economic Evaluations (http://joannabriggs.org/assets/docs/critical-appraisal-tools/JBI_Critical_Appraisal-Checklist_for_Economic_Evaluations.pdf) [31], adapted from the Drummond checklist [32], which addressed: the study question; description of alternatives; identification of costs and outcomes; establishment of clinical effectiveness; accuracy, credibility and timing of costs and outcomes; incremental analysis; sensitivity analyses; and generalizability. Studies were rated as ‘yes’, ‘no’, or ‘unclear’ in terms of their compliance with each quality criterion in light of the objective of the study. For instance, a study which was designed to focus only on costs would by definition not have considered outcomes and so it may still score a ‘yes’ on item 3 which considers whether all relevant costs and outcomes have been identified. A study which was designed as a full economic evaluation on the other hand would need to identify both costs and outcomes to meet this criterion. As the search strategy did not impose date limits, the purpose of this appraisal was not to exclude studies that pre-dated the use of current economic evaluation methods. Rather the purpose of appraisal was to identify methodological issues with the study design that may result in biased measures of cost and/or effect in order to inform the interpretation of study results.

The JBI Critical Appraisal Checklist for Economic Evaluations was chosen for the current study as it covers the same ten items as the Drummond checklist with the addition of an eleventh item which addresses the generalizability of results to the setting of interest for the review [31]. The appraisal was conducted by the primary author (T.E.) and ratified by a second reviewer (R.M.). Disagreements were resolved through discussion between the primary and secondary reviewer.

### Data synthesis

Data extracted from included studies were analysed and synthesized in a narrative summary to address the stated review objective. Synthesis included (1) key findings pertaining to organisational and environmental characteristics aimed at improving the quality of care for older adults in a residential aged care setting; (2) a review of approaches taken to include health and quality of life effects in the identified economic analyses; (3) a review of approaches taken to include residents with dementia in the identified economic analyses; and (4) consideration of key methodological issues for consideration in the future design and conduct of economic evaluations of residential aged care infrastructure. This review was prepared in accordance with the Preferred Reporting Items for Systematic Reviews and Meta-Analyses (PRISMA) statement [33].

## Results

### Search and study selection

**Fig. 1:**
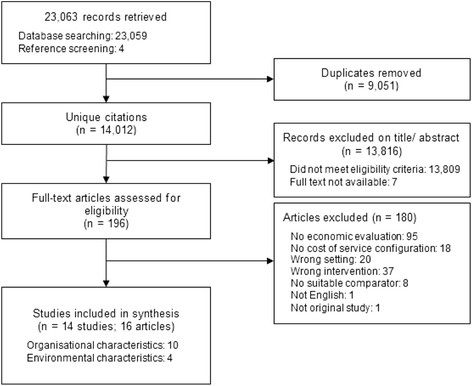
Flow diagram of study selection

The study selection process is presented in Fig. 1. The electronic database search yielded a total of 23,059 citations; an additional 4 citations were identified through searches of reference lists of included studies. A total of 14,012 unique citations were identified after duplicate removal. After title and abstract screening 13,809 records did not meet eligibility criteria and 7 studies were excluded as the full texts were not available. Full text reviews were conducted for 196 articles and 14 studies, from 16 publications, met the inclusion criteria. The unit of analysis for the purpose of this review was the study, rather than individual publications. We report the findings of this review in accordance with the Preferred Reporting Items for Systematic Reviews and Meta-Analyses (PRISMA) statement [33]. The completed PRISMA checklist is included in Additional file 1.

### Overview of studies

Table 1 presents the main characteristics of studies included in the review. All 14 studies contained a partial economic evaluation in the form of a cost analysis. None of the identified studies undertook a full economic evaluation in the form of a cost-benefit, cost-effectiveness, or cost-utility analysis. The majority of studies (*n* = 13) were evaluated from an institutional perspective, and only costs occurring within the facilities themselves were considered. Two of the studies were specific to residents with dementia, in which all residents participating in the study had a recorded diagnosis [34, 35].

**Table 1:** Characteristics of included studies

Source, Country	Interventions/Comparator^a^	Facility n	Participant n	Study design^a^	Type of economic evaluation; analytic viewpoint	Date/source/currency of economic data^a^	Dementia specific	Setting^a^
Studies of Organisational Characteristics (*n* = 10)								
Arling 1987 [44] United States	Chain-affiliated Independent for-profit Government-owned/not-for-profit	150	N/A	Cross-sectional	Cost analysis; Institutional	1984–1985 ; Medicaid cost reports; *USD*	No	ICF
Bland 1992 [37] United Kingdom	Government-owned For profit Not-for-profit	Phase 1: 100 Phase 2: 6	Phase 1: 2,405 Phase 2: 156	Cross-sectional	Cost analysis; Institutional	1990; Annual reports, accounts, and individual financial returns; *GBP*	No	RH
Davis 1993 [41] United States	For profit Not-for-profit	178	N/A	Cross-sectional	Cost analysis; Institutional	1989; Medicaid Certification inspection surveys and cost reports; *USD*	No	NH
Farsi 2004 [36] Switzerland	Government-owned Not-for-profit	36	N/A	Cross-sectional time series	Cost analysis; Institutional	1993–2001; Annual accounting reports converted to year 2000 Swiss Francs; *CHF*	No	NH
Holmes 1996 [45] United States	Chain-affiliated Proprietary non-chain Freestanding not-for-profit Government-owned Hospital-based	393	N/A	Cross-sectional	Cost analysis; Institutional	1989; Medicaid Certification inspection surveys and cost reports; *USD*	No	NH
Smith 1992 [50] United States	Rural location Urban location	52	N/A	Cross-sectional	Cost analysis; Institutional	1987–1988; Medicaid cost reports; research questionnaire to gather performance, strategy, and strategic planning data; *USD*	No	NH
Sulvetta 1986 [49] United States	Hospital-based Freestanding	3,492	N/A	Cross-sectional	Cost analysis; Institutional	1980; Medicare cost reports, projected to 1983; *USD*	No	SNF
Ullmann 1984 [46] United States	For profit Not-for-profit Government-owned	308	N/A	Cross-sectional	Cost analysis; Institutional	1977; National Nursing Home Survey; *USD*	No	SNF
Ullmann 1986 [48] United States	Independent Chain-affiliated	265	N/A	Cross-sectional	Cost analysis; Institutional	1977; National Nursing Home Survey; *USD*	No	SNF
Ullmann 1987 [47] United States	For profit Not-for-profit Government-owned	494	N/A	Cross-sectional	Cost analysis; Institutional	1976; Source not stated; *USD*	No	SNF
Studies of Environmental Characteristics (*n* = 4)								
Calkins 2007 [38] United States	Private room s Enhanced shared rooms Traditional shared rooms	N/A	189 (bedrooms)	Cross-sectional	Cost analysis; Institutional	Date not disclosed.; Standard commercial-grade-construction assumptions for the Cleveland, Ohio area; *USD*	No	NH
Chenoweth 2014 [40] Australia	Person-centred care (PCC) Person-centred environment (PCE) Both PCC + PCE Usual care	38	601	Cluster Randomised Controlled Trial	Cost analysis; Institutional	2009–2011; Resource use measured and unit costs assigned using market rates; *AUD*	Yes	RACF
Jenkens 2011 [39] United States	Green House model Usual care	7	N/A	Cross-sectional	Cost analysis; Institutional	2008; Commercial construction costs sourced from Reed Construction Data; *USD*	No	SNF
Maas 1998 [35]; Swanson 1993 [42]; Swanson 1994 [43] United States^b^	Special care unit Traditional unit	1	44	Prospective cohort study	Cost analysis; Health care	Date not disclosed; Resource use measured and unit costs assigned - source of unit cost data not disclosed; *USD*	Yes	NH

Settings: ICF, intermediate care facility; SNF, skilled nursing facility; NH, nursing home; RACF, residential aged care facility; RH, residential home

^a^Headings taken from the JBI Data Extraction Form for Economic Evaluations (http://joannabriggs.org/assets/docs/jbc/operations/dataExtractionForms/JBC_Form_DataE_EconEval.pdf)

^b^All three publications are from the same study

Ten of the studies evaluated specific organisational characteristics, while four focused on environmental characteristics. The most frequent study design was cross-sectional (*n* = 11). Other study designs included a cluster-randomised controlled trial (*n* = 1), cross-sectional time series (*n* = 1), and prospective cohort (*n* = 1). Twelve studies pertaining to organisational characteristics were undertaken in the United States with cost data from large data sets collected during the 1970s and 1980s. Only two studies were conducted outside the United States: one study conducted in Switzerland using cost data for the period 1993–2001 [36] and one study conducted in the United Kingdom during 1990–1992 [37]. Three of the studies evaluating environmental characteristics were conducted in the United States [35, 38, 39], while the fourth was conducted in Australia [40].

The number of participating facilities per study ranged from 1 to 3,492 (mean: 424; median: 150). Of the three studies that recruited resident participants, sample sizes varied widely (*n* = 44 [35]; *n* = 601 [40]; *n* = 2,405 [37]). The 11 studies that did not recruit resident participants collected facility-level data only, such as operating costs or staff time. Clinical outcome measures - defined as outcomes involving measurable changes in a resident’s health or quality of life - were reported in 3 studies (across 4 articles) [40–43]. A summary of main clinical outcomes for the 3 studies are reported in Table 2 and include measures of agitation, quality of life, social interactions and behaviour, cognitive status, function, and a composite measure of poor quality based on rates of decubitus ulcers, catheterization, physical restraints, chemical restraints, and drug errors.

**Table 2:** Summary of clinical outcomes, measurement instrument used, and effectiveness for studies reporting a clinical outcome

Intervention, Study	Outcome	Measurement instrument	Effectiveness results as measured by measurement instrument (baseline; follow-up)
Proprietary status, Davis 1993 [41]	Composite measure of poor quality based on rates of decubitus ulcers, catheterization, physical restraints, chemical restraints, and drug error rates.	Composite index *Higher scores indicate poorer quality*	*Mean (SD)* For-profit: 0.34 (2.30) Not-for-profit: −0.57 (1.87)
Person-centred environment (PCE), Chenoweth 2014 [40]	Agitation	Cohen Mansfield Agitation Inventory (CMAI) *Higher score indicates greater agitation*	*Mean (95% CI)* Control: 52 (43–61); 51 (41–62) PCE: 65 (57–73); 55 (46–64); *p* = 0.04
	Quality of life	DemQol-proxy *Higher score indicates improved quality of life*	*Mean (95% CI)* Control: 101 (98–104); 103 (99–106) PCE: 101 (99–104); 106 (103–109); *p* = 0.02
	Social interactions and behaviour	Care interaction quality (QUIS) *% interactions positive*	*Mean (95% CI)* Control: 78 (73–83); 82 (76–88) PCE: 78 (74–83); 82 (76–87); *p* = 0.55
		Emotional responses in care (ERIC) *% positive*	*Mean (95% CI)* Control: 25 (20–30); 25 (18–31) PCE: 23 (18–28); 26 (21–32); *p* = 0.63
Special care unit (SCU), Swanson 1993 [42] Swanson 1994 [43]	Cognitive status	Alzheimer’s Disease Assessment Scale (ADAS) *Cognitive dimension, higher scores indicate lower cognitive ability*	*Mean (SD)* Traditional: 45.38 (15.64); 52.88 (17.89) SCU: 56.67 (12.94); 59.69 (12.95)
	Social interactions and behaviour	Individual Incident Reports (IIR) *Number of catastrophic reactions*	*Mean (SD)* Traditional: 82; 46 SCU: 156; 48
	Function	Functional Abilities Checklist (FAC) *Higher score indicates greater function*	*Mean (SD)* Traditional: 73.67 (15.41); 71.63 (12.25) SCU: 76.15 (12.35); 76.23 (9.76)
		Geriatric Rating Scale (GRS) *Higher score indicates reduced function*	*Mean (SD)* Traditional: 30.89 (8.18); 35.13 (10.22) SCU: 32.69 (7.76); 35.39 (7.38)

### Organisational characteristics

Interventions reported in studies pertaining to organisational characteristics fell into four broad categories: proprietary status, affiliation, size, and location.

#### Proprietary status

Of the seven studies that focused upon proprietary status, six compared for-profit facilities to one or more alternative proprietary status, and all studies indicated that for-profit facilities provided care at the lowest cost [37, 41, 44–47]. One study compared private not-for-profits to public (i.e. government-owned) not-for-profits and found no significant cost differences [36]. In three of the studies, clinical and process-related outcomes were utilized as markers for the quality of care provided [41, 45, 46]. These proxy measures of care quality varied widely and included rates of decubitus ulcers, catheterisation, physical restraints, chemical restraints, drug error, number of regulatory deficiencies, skill level of persons in charge of nursing shifts, range of therapies provided, and number of people waitlisted. One study sourced quality measures from a state-wide composite rating scale which combined three quality assessment tools administered by interdisciplinary survey teams to evaluate compliance with the state hospital code, federal regulations, and individual resident medical reviews [47] to give an overall rating of either “very good”, “good”, “needs improvement”, or “unsatisfactory”. Results indicated a distinct lack of variation amongst the quality ratings for the 494 facilities included in the study, with over 95% of facilities receiving a rating of “good” for overall quality.

A study by Bland and colleagues [37] attempted to link costs to quality across Scottish residential homes for older people in three sectors: public (government-owned), for-profit and not-for-profit. The study concluded that there were no readily identifiable patterns of trade-offs between cost and quality across the three sectors. However, through a comparison of operating costs, the study suggested that the for-profit sector was a low-cost operator, the not-for-profit sector operated in the mid-range for costs, and the public sector operated at the highest cost. Analysis of quality of care data found that larger facilities (within respective sectors) and government-owned facilities (between sectors) were associated with better care. Quality of care was assessed on 130 primary variables through a combination of interview with the facility’s officer-in-charge and researcher observation. The quality of care scale was classified into 8 groups: building; procedures; regime; medical care; promotion of continence; care of dementia sufferers; general services; and interviewer-observation.

#### Affiliation, size and location

Affiliation refers to both hospital-based facilities and facilities owned as part of a chain, as compared with freestanding or independent facilities. Freestanding facilities are those which are not part of a hospital. Independent facilities are those which are not affiliated with a chain. Chain affiliation is defined as membership in a group of facilities operating under one authority or ownership. The minimum number of facilities required to meet this definition varied between studies ranging from three or more facilities [44] to five or more facilities [45] while a third study did not specify a particular number [48]. While there is some evidence to suggest hospital-based facilities have relatively lower capital costs compared with non-hospital based (freestanding) facilities [45], both operating costs and total costs were found to be higher in hospital-based facilities when compared with freestanding institutions [45, 49]. Three studies evaluated the effects of chain-affiliations on operating and total costs, with conflicting results reporting chain-affiliated facilities as having no difference in costs [44], higher costs [45], and lower costs [48] when compared with independent facilities.

Out of four affiliation studies identified by this review [44, 45, 48, 49], process-related outcome measures designed to give an indication of the quality of care provided at the facility were examined in three of the studies and included number of regulatory deficiencies [45], presence of rehabilitation services and nurse-to-bed ratios [49], and facility age, number of therapies provided, and the facility’s wait-list size [48]. Chain and free-standing facilities had the highest average annual deficiencies, while hospital-based facilities had the least [45]. Hospital-based facilities were also found to have more rehabilitation services and higher nurse-to-bed ratios [49], which was suggested to indicate higher quality care.

One study compared rurally located nursing homes (*n* = 34) with urban-based facilities (*n* = 18) and found no significant cost differences [50]. No effectiveness data was collected; rather comparisons were made based on facility profits. A second study reported urban-based facilities as having higher total costs than rural facilities [49]. Process-related outcome measures of quality in this study found rural facilities to have higher nurse-to-bed ratios but fewer rehabilitation services.

Only one study was identified which specifically focused on the costs associated with the size of facility. Marginally lower average costs were reported for facilities with 100–199 beds compared with 0–49 beds, 50–99 beds, and 200 or more beds [46]. No effectiveness or quality data were reported.

### Environmental characteristics

Interventions reported in studies pertaining to environmental characteristics fell into two broad categories, functional modifications and home-like environments.

#### Functional modifications

Two studies examined the effects of functional modifications on residents with dementia. Interventions consisted of adjustments to existing spatial configurations with the aim of improving the safety, accessibility and utility of both indoor and outdoor spaces. One study undertook a cluster-randomised controlled trial examining the effects of both person-centred care and person-centred environments for residents with dementia [40]. Modifications varied between participating facilities (*n* = 38), and included changes such as extending activity spaces, modifying internal walls to increase visual access to bedrooms and activity spaces, increasing ease of access to courtyards and gardens, building partitions to reduce overstimulation in large group spaces, and improving gardens and landscaped exteriors with paving, new sitting areas, and covered spaces. It was estimated that all environmental modifications (implemented between 2009 and 2011) cost less than 10,000 Australian Dollars per facility to implement, with the average facility spending 9,198 Australian Dollars.

Outcome measures collected included: quality of life (DemQol and DemQol-proxy), agitation (CMAI - Cohen Mansfield Agitation Inventory), emotional responses (Emotional Responses to Care instrument), and quality of care interactions (QUIS instrument). Results for outcome measures were inconsistent although small statistically significant improvements were found for some participants in levels of agitation, with CMAI scores decreasing from 65 pre-intervention to 55 at the 8-month follow-up in the environmental intervention group compared with the control group which reported CMAI scores of 52 and 51 at pre-intervention and follow-up respectively (*p* = 0.04) [40].

A cost analysis of special care units (SCUs) for residents with dementia conducted by Maas and colleagues [35] provided data on SCU construction and remodelling costs. In this study, participants with dementia were matched by age and cognitive function, and randomly assigned to the SCU or one of the traditional units at the same facility. Modifications to the SCU included redecorating, door modifications and installation of a security system, new walls in the lounge and dining areas, bedroom privacy curtains and special furniture, and installation of a fence in the outdoor area. Total remodelling costs on the SCU (home to 37 residents) were 89,700 US Dollars (date of cost data unknown).

Effectiveness measures from the SCU study were examined in two additional publications [42, 43]. Primary outcome measures included cognitive status (Alzheimer’s Disease Assessment Scale) and functional abilities (Functional Abilities Checklist and the Geriatric Rating Scale). No significant differences in cognition or function were found between residents on the SCU, and those in the traditional units [43]. However, the number of catastrophic reactions reduced significantly on the SCU compared with traditional units with the number of reactions decreasing from 156 pre-intervention to 48 at the 12-month follow-up in the SCU group compared with the control group which reported catastrophic reactions of 82 and 46 at pre-intervention and follow-up respectively (*p* = 0.035) [42]. A catastrophic reaction in dementia is defined as an excessive reaction to a seemingly normal, non-threatening situation (e.g. a question asked of the person, bathing, dressing) and is characterised by mood changes or reactions such as weeping, blushing, anger, or agitation [42]. Catastrophic reactions were recorded for each resident on an Individual Incident Record by nursing staff.

#### Home-like environments

Two studies examined costs associated with providing more home-like care environments. An analysis of bedroom plans conducted by Calkins and Cassella [38] examined room size and construction cost differences between private rooms, shared rooms, and enhanced shared rooms (designed to give the resident a well-defined and generally exclusive territory within the shared room). Findings indicated that private rooms cost more to construct on a per resident basis than shared or enhanced shared rooms. No quantitative quality measures were included in the study. Rather the authors examined the effectiveness of private rooms through a systematic review, interviews and focus groups, the results of which indicated better outcomes associated with private rooms, with evidence indicating that older adults have a strong preference for private bedrooms [38, 51]. Clinical outcomes associated with private rooms, identified as part of the authors’ systematic review, included reduced risk of infection such as influenza and gastroenteritis [52, 53].

One study examined the Green House model, which is an alternative living environment to the traditional skilled nursing facilities in the United States that aims to provide a more person-centred, consumer-driven environment. In the Green House model, ten to twelve residents live in a self-contained residence designed to look and feel like a private home. Each resident has a private bedroom and bathroom, and each residence has an open kitchen, living room, and dining room, as well as access to outdoors through a patio or balcony. An analysis of capital costs conducted by Jenkens and colleagues [39] concluded that the Green House model incurred slightly higher capital costs than traditional skilled nursing facilities largely as a result of the increased square foot requirements (an additional 300 square feet per resident, on average). Facility type, size, labour rates, and site-specific preparation costs were found to be the primary drivers of capital costs. No quality or outcome measures were included alongside the measurement of costs assessed in this study.

### Critical appraisal

Table 3 presents the results of the assessment of methodological quality of the included studies. The methodological quality of included studies varied widely and a high level of uncertainty was found in the generalisability and transferability of findings. The primary methodological issues identified included: a failure to establish clinical effectiveness in any of the studies, the absence of incremental analysis of costs and consequences in all studies, and a lack of sensitivity analyses to assess the robustness of the base case results to variations in key parameters. Although no studies established clinical effectiveness, two studies (in three articles) did provide effectiveness estimates for the interventions conducted [40, 42, 43], while eight studies utilized clinical or process-related outcomes or observable qualities such as staff-to-resident ratios as markers for quality of care [36, 37, 41, 45–49].

**Table 3:** Critical appraisal results for included studies using the JBI Critical Appraisal Checklist for Economic Evaluations

Source	Q1	Q2	Q3	Q4	Q5	Q6	Q7	Q8	Q9	Q10	Q11
	Well-defined question	Comprehensive description of alternatives	All important and relevant costs and outcomes for each alternative identified	Clinical effectiveness established	Costs and outcomes measured accurately	Costs and outcomes valued credibly	Costs and outcomes adjusted for differential timing	Incremental analysis of costs and consequences	Sensitivity analyses conducted	Study results include all issues of concern to users	Results are generalizable
Arling 1987 [44]	Yes	Yes	Yes	No	Unclear	Yes	No	No	No	Yes	No
Bland 1992 [37]	Yes	Yes	Yes	No	Yes	Yes	No	No	No	Yes	Unclear
Davis 1993 [41]	Yes	Yes	Yes	No	Yes	Yes	No	No	No	Yes	No
Farsi 2004 [36]	Yes	Yes	Yes	No	Yes	Yes	No	No	No	Yes	Unclear
Holmes 1996 [45]	Yes	Yes	Yes	No	Yes	Yes	No	No	No	Yes	No
Smith 1992 [50]	Yes	Yes	Yes	No	Yes	Yes	No	No	No	Yes	Unclear
Sulvetta 1986 [49]	Yes	Yes	Yes	No	Yes	Yes	No	No	No	No	Unclear
Ullmann 1984 [46]	Yes	Yes	Yes	No	Yes	Yes	No	No	No	Yes	Unclear
Ullmann 1986 [48]	Yes	No	Yes	No	Yes	Yes	No	No	No	Yes	Unclear
Ullmann 1987 [47]	Yes	Yes	Yes	No	Unclear	Yes	No	No	No	Yes	Unclear
Calkins 2007 [38]	Yes	Yes	Yes	No	Yes	Unclear	No	No	No	No	Yes
Chenoweth 2014 [40]	Yes	Yes	No	No	Unclear	Yes	No	No	No	No	Unclear
Jenkens 2011 [39]	Yes	Yes	Yes	No	Yes	Yes	No	No	Yes	Yes	Yes
Maas 1998 [35]; Swanson 1993 [42]; Swanson 1994 [43]	Yes	Yes	Yes	No	Unclear	Unclear	No	No	No	No	Unclear

Further methodological issues were identified in relation to the reporting of resource use and costs. Four studies reported mean costs but did not provide a measure of variation or dispersion in the cost results (e.g. standard deviation) [35, 38, 40, 49], two studies did not fully disclose the source of their cost data [35, 47] and two studies did not disclose the date for their cost data collection [35, 38]. Out of ten studies addressing organisational characteristics, only one study reported on resource use, reporting mean staff time per resident per week [37]. Similarly, of the four studies relating to environmental characteristics, only one study reported resource use which was reported in the form of room size measurements [38].

## Discussion

A total of 14 studies pertaining to organisational and environmental characteristics in residential care were identified by this review, all of which contained partial economic evaluations in the form of cost analyses. The quality of study designs varied across the included studies, and as such study results should be treated with caution. Eight studies utilized various clinical or process-related outcomes as proxies for the quality of care provided, and two studies focused on resident outcomes including agitation, quality of life, and the quality of care interactions. However none of the identified studies attempted to systematically link costs and outcomes in the form of a cost-benefit, cost-effectiveness, or cost-utility analysis. The majority of studies (*n* = 12) did not specifically highlight organisational and/or environmental characteristics pertaining to residents living with dementia.

Formalising these issues within the framework of a systematic review has highlighted the paucity of evidence in this area. The usefulness of studies containing only partial economic evaluations is limited for policy and decision makers, in that they do not present the case on whether the costs of a course of action is worthwhile in terms of benefits provided to improve quality of care, leaving this aspect up to the reader to decide. The studies identified by this review provide a starting point from which to develop future economic studies and the methodological issues discussed throughout this section emphasize the need to do a better job of collecting and reporting data that is helpful for decision makers.

### Key findings pertaining to organisational and environment characteristics

In terms of organisational factors, the available literature suggests that for-profit facilities operate at lower costs than not-for-profit and government-owned facilities, while hospital-based facilities may have lower running costs than free-standing facilities. It is important that these results be interpreted with caution firstly because the cost data presented in these studies are dated, having been collected between 1976 and 1989. Secondly, all but one of the studies addressing proprietary status and affiliation were conducted in the United States and therefore their transferability to other aged care systems around the world is unclear. That being said, the value of investigating the cost-effectiveness of organisational characteristics should not be dismissed. While the evidence pointing to cost differences may be dated, there is current literature which identifies variation in outcomes based on organisational factors. For instance, for-profit facilities have been associated with higher staff turnover [54, 55], lower nursing staff levels [55], and lower quality care overall [56]. Given the available literature indicating differences in both costs and effectiveness, future research which aims to link quality measures with cost data for differing proprietary status may provide insight into questions such as whether additional resources allocated in a not-for-profit organisation are producing better outcomes, or if perhaps these organisations are operating less efficiently.

There is a paucity of evidence regarding the impact of location or size on the running costs and cost-effectiveness of residential care facilities. Our review found only two studies related to locality and one study which investigated facility size and thus it is difficult to draw conclusions. There have been a number of studies, however, which have looked at associations between these organisational factors and clinical outcomes. For instance, in a study investigating the use of feeding tubes among residents with advanced cognitive impairment, residents living in urban facilities and residents living in facilities with more than 100 beds were found to have an increased likelihood of having a feeding tube despite empirical data suggesting that feeding tubes are not beneficial in this population [57]. Facilities with more than 100 beds have also been linked to higher staff turnover which has been found to be detrimental to overall quality of care [55]. In light of evidence which links quality outcomes to size and location, future economic evaluation studies are warranted.

The body of evidence examining the impact of the physical environment on people with dementia has been well documented, and environmental design interventions have been shown to affect behaviour, function, well-being, social abilities, orientation, and care outcomes [14]. SCUs have been linked to lower hospitalisation rates [58] and lower likelihood of using feeding tubes [57]. However, economic evaluations of environmental characteristics and dementia-specific facility designs are scant; our review identified only four studies in this domain. Environmental modifications in the identified studies included homelike environments (e.g. single bedrooms, private bathrooms, decorating, and access to outdoors) and functional modifications (e.g. increasing visual access to bedrooms and activity rooms, extending activity spaces, and building partitions to reduce overstimulation). The economic evidence in this review indicates that environmental modifications come at an additional cost, but are weakly associated with better outcomes in the form of reduced agitation and improved social interactions. It is important for future studies investigating the effectiveness of a particular environmental intervention to conduct economic evaluations alongside these trials in order to build a more robust evidence base surrounding the value of investing in specialised designs.

### The inclusion of health and quality of life effects

One very prominent methodological issue that emerged from this review was the heterogeneous range of outcomes that have been used. Some of the direct outcomes measured included agitation, improved social interactions, quality of life, behaviour, function, well-being, depressive symptoms, quality of care, rates of decubitus ulcers, catheterisation, physical restraints, and chemical restraints. Other outcomes, which could be presumed to impact on health, included drug errors, number of regulatory deficiencies, skill level of persons in charge of nursing shifts, range of therapies provided, and number of people waitlisted. The development of guidance towards a more consistent methodology for economic assessment of residential aged care infrastructure is needed, specifically with the inclusion, where possible, of the health and quality of life benefits measured from the perspective of the residents themselves including people with dementia.

There have been numerous instruments developed to measure health benefits such as behaviour, function, well-being, care outcomes, and health-related quality of life, for example. Consequently, it is important for the chosen outcome to be an appropriate measure of achievement for the desired objective. For instance, the desired objective of aged care infrastructure may be to improve the quality of life for the residents who live there. The question then becomes what is the most reliable outcome measure to capture improvements in the lives of residents?

One approach may be to present an array of outcome measures for each alternative, allowing the decision-makers to make their own trade-offs between measures of effectiveness. This is commonly known as a cost-consequences analysis. Another possibility is incorporating a generic measure of incremental benefit, such as the QALY. The main benefit of utilising QALYs in this context would be their applicability to all aged care residents, which would allow decision makers to make comparisons across differing programs. Cost-utility analyses, which use QALYs as the outcome measure, are the recommended economic evaluation in national guidelines developed by government agencies in healthcare such as NICE in the UK [21], and the Canadian Agency for Drugs and Technologies in Health in Canada [59]. While these guidelines were developed for economic evaluations of health technologies, they could potentially be applied to aged care infrastructure, for instance where meaningful differences in health-related quality of life between the intervention and comparator have been demonstrated.

It may also be worthwhile to consider a social context, rather than a health context, as potentially more appropriate in a residential care setting. Current research has acknowledged factors outside of health status such as dignity, independence, and having control over their daily lives as important contributors to residents’ quality of life [60, 61]. A recent systematic review of instruments for measuring outcomes in economic evaluations within aged care recommends the use of a generic preference based measure of health related quality of life such as the EQ-5D to obtain QALYs in combination with an instrument with a broader quality of life focus to capture dimensions of social well-being, such as the Adult Social Care Outcomes Toolkit (ASCOT) designed to evaluate interventions in social care, or the ICEpop CAPability measure for Older people (ICECAP-O) which measures capability in older people [62]. Ultimately, it is important that the chosen method is sensitive enough to measure changes for this population, and broad enough to allow comparisons to be made at a service planning level.

### The inclusion of residents with dementia

Twelve studies identified by this review did not disclose whether residents with dementia had participated. While it is uncertain whether these studies included participants with dementia, the omission suggests that no consideration was given to this subgroup during study design. One study specified that residents were only approached to participate if judged by staff to be capable of self-completing the study questionnaire [37], which suggests cognitively impaired residents were excluded. When designing economic evaluations, it must be ensured that the study sample is representative for the population being assessed. The quality of an economic evaluation is highly dependent on the source of data used, and its ability to be transferred to other settings. In residential care settings, the exclusion of residents with dementia raises serious concerns regarding the representativeness of data given that over 50% of those residing in aged care facilities have a recorded diagnosis of dementia [7, 9].

### Further methodological issues

In addition to the issues discussed surrounding the measurement of health and/or quality of life effects, and increasing the representativeness of data by ensuring the inclusion of residents with dementia, an important methodological issue to consider is study design. The common methodology used in the health care sector for implementation research is a cluster randomised design, as participant-level randomisation can introduce bias through exposure of the control group to the intervention [63]. Only one of the studies identified used a cluster randomised design [40]. Employing a randomised design to focus upon the impact of organisational characteristics is often not achievable in the aged care sector. It is not feasible to randomize attributes such as the proprietary status or location of an aged care facility. As shown by this review, observational study designs are much more practical in this setting. However, a cross-sectional study design, which was the most frequently used design in included studies, can identify associations but not causality due to the absence of a time dimension. Well-designed observational studies with a temporal dimension (i.e. prospective or retrospective rather than cross-sectional) have been shown to produce comparable results to randomised controlled trials [64, 65].

An alternative option when randomised controlled trials are not feasible or for extrapolating beyond the time frame of a clinical trial is decision modelling [66, 67]. Using a decision modelling approach, costs and outcomes can be predicted using data synthesised from disparate sources and models can be built to extrapolate long term estimates of costs and benefits. While none of the studies identified in this review utilised a decision modelling approach, this may be a viable direction for future research [67].

Transparency in reporting study methods and results is another area that is important when assessing the validity and reliability of economic evaluations. This is not specific to residential care or to infrastructure, but nonetheless an important consideration. A clear example is the cost analysis of special care units published in 1998 by Maas and Buckwalter [35] which failed to disclose the date the cost data was collected or whether costs were adjusted for inflation. The exact date of this study was not stated, though the first preliminary results were published in 1988 [68], 10 years prior to the cost analysis publication. Future economic evaluations in this area should strive to meet the quality standard for reporting economic evaluation as specified in the Consolidated Health Economic Evaluation Reporting Standards (CHEERS) statement [69] including the quantities of resources used in addition to costs and incorporating the measurement and valuation of service outcomes and quality of life. Disclosures should also be included to indicate the timing of cash flows and the sources of cost data.

### Strengths and limitations of the review

This systematic review had a broad scope in order to provide a comprehensive summary of the evidence, and as such we can be confident that we have captured the majority of studies on this subject. The main strength of this review was the systematic and transparent approach which, in combination with the breadth of the objective, allowed for a thorough synthesis of existing economic evaluations of residential aged care infrastructure. The review was conducted to a high methodological standard and met the quality standards set within the Preferred Reporting Items for Systematic Reviews and Meta-Analyses (PRISMA) statement. Critical appraisal of studies was undertaken using the JBI Critical Appraisal Checklist for Economic Evaluations which is a well-recognised and highly regarded Checklist for assessing the quality of economic evaluation studies previously utilised in other high quality systematic reviews published previously [70, 71]. However, the broad scope of this review, and the incorporation of economic evidence meant that it was necessarily time-intensive, requiring more resources for the search process, data extraction, and analysis compared with a narrow scope review. For pragmatic reasons, one author took responsibility for both the initial examination of all citations and for all data extracted from included studies, and as such it is possible that errors occurred. This review had limitations to analysis imposed by the heterogeneity of interventions, methods, and outcomes in the included studies. A meta-analysis was not possible; rather the review relied on a narrative analysis of the included studies. This is a reflection of the research that has been conducted to date, and again highlights the need for future evaluation research to be carefully planned such that the data collected and reported is useful for decision makers.

## Conclusions

This research highlights a gap in economic evidence, and this evidence is needed to inform future aged care sector facility design and development. Despite the high cost of providing care to older people in residential care facilities, there is a lack of robust economic evidence on the value of organisational and environmental design features. There is a shortage of research linking costs to outcomes. The quality of existing cost analyses and economic evidence is varied, and much of the existing research is outdated which limits the usefulness of the data.

Key methodological issues for consideration in the design of economic evaluations of residential care infrastructure include robust study designs, valuing health and/or quality of life effects in a meaningful way, and increasing the representativeness of data by ensuring the inclusion of residents with dementia.

Future research should focus on identifying appropriate and meaningful outcome measures that can be used at a service planning level, as well as the broader health benefits and cost-saving potential of different organisational and environmental characteristics in residential care.

## Additional file

Additional file 1: PRISMA checklist (DOC 63 kb)

## Abbreviations

ADAS: Alzheimer’s Disease Assessment Scale
ASCOT: Adult Social Care Outcomes Toolkit
AUD: Australian Dollar
CHEERS: Consolidated Health Economic Evaluation Reporting Standards
CHF: Swiss Franc
CINAHL: Cumulative Index of Nursing and Allied Health Literature
CMAI: Cohen Mansfield Agitation Inventory
ERIC: Emotional Responses in Care
FAC: Functional Abilities Checklist
GBP: British Pound
GDP: Gross domestic product
GRS: Geriatric Rating Scale
ICECAP-O: ICEpop CAPability measure for Older people
ICER: Incremental cost-effectiveness ratio
ICF: Intermediate care facility
IIR: Individual incident reports
JBI: Joanna Briggs Institute
NH: Nursing home
NHMRC: National Health and Medical Research Council
OECD: Organisation for Economic Co-operation and Development
PCC: Person-centred care
PCE: Person-centred environment
PRISMA: Preferred Reporting Items for Systematic Reviews and Meta-Analyses
QALY: Quality-adjusted life year
QUIS: Quality of care interactions
RACF: Residential aged care facility
RH: Residential home
SCU: Special care units
SNF: Skilled nursing facility
USD: United States Dollar
